# Comparison of Thermal Behaviors of Carbon and Stainless Steel Billets during the Heating Process

**DOI:** 10.3390/ma17010183

**Published:** 2023-12-28

**Authors:** Joong-Ki Hwang

**Affiliations:** School of Mechatronics Engineering, Korea University of Technology & Education, Cheonan 31253, Republic of Korea; jkhwang@koreatech.ac.kr; Tel.: +82-041-560-1642

**Keywords:** thermal conductivity, latent heat, billet, thermal behavior, heating process

## Abstract

The effect of thermal properties on the thermal behavior of a steel billet was investigated during the heating process to understand each effect and to provide process engineers with sufficient data to choose the optimal design conditions in reheating the furnace for hot rolling. Carbon steel and austenitic stainless steel (STS) were compared based on numerical simulations owing to the completely different thermal properties of these two steels: carbon steel having a phase transformation with a relatively high thermal conductivity and STS having no phase transformation with a relatively low thermal conductivity. The thermal conductivity affected the thermal behavior of the billet in the initial stage of heating owing to the high temperature difference between the surface of the billet and the gas in the furnace, i.e., the high Biot number. Accordingly, a non-firing zone and/or a preheating zone with a low gas temperature are necessary for high-alloyed steels including STS because the thermal conductivity of these steels is relatively low. The phase transformation of the carbon steels needs to occur in the primary heating zone, and this zone needs to have a relatively low gas temperature to reduce the temperature deviation or thermal stress in the billet. The heating pattern of the carbon steels and STSs in the reheating furnace should be designed differently considering the thermal conductivity and latent heat by the phase transformation of steels to obtain a high heating quality for the billet.

## 1. Introduction

The main role of the reheating furnace in a hot rolling mill is to achieve the desired billet target temperature with acceptable uniformity along the width, thickness, and length directions of the billet with minimum energy consumption [1,2]. Industrially, it is judged that the target temperature has been achieved when the billet reaches the designed temperature on average within a given temperature deviation. The temperature prediction models are generally used in the reheating furnace for hot rolling to accurately predict this target temperature and temperature deviation within the region [3,4,5] because it is difficult to measure both the temperature of a billet and the temperature deviation of a billet within a region. The target temperature and temperature deviation within the billet are designed to ensure rolling quality. An incorrectly designed target temperature and temperature deviation within the billet increases the rolling force, causes surface defects in hot-rolled products, and causes the product to exhibit undesirable shape, microstructure, and mechanical properties after rolling. Accordingly, the industrial rolling mill tracks the temperature distribution of the billet during the entire heating process based on thermal prediction models for the billet. The thermal model predicts the temperature of a billet based on the measured gas temperature using thermocouples installed in a reheating furnace. In other words, the thermal prediction model calculated the temperature of a billet using the gas temperature in a reheating furnace from the charging to the discharging of a billet.

In the steel rolling industry, various steel grades are heated in the furnace for hot rolling on an hourly basis. In particular, the steel grades change more frequently during the billet rolling process than during the slab rolling process because the wire, rod, and bar products have the characteristics of small-lot productions of various types. Some of the billets are warm-charged, whereas other billets are cold-charged to the reheating furnace. In addition, the target heating temperature varies over a wide range, i.e., 900–1300 °C, depending on the mechanical properties and chemical compositions of the initial billet, the reduction ratio during rolling, the desired microstructures of the final product, and the surface properties of the product such as surface defects, decarburization depth, and oxide scale. Overall, the industrial billet reheating process is complicated for mass production, making it difficult to ensure a target temperature with acceptable uniformity and energy consumption. Therefore, the better scheduling of the hot rolling mill including a reheating furnace can improve the productivity of the mill, the product quality, the energy efficiency, and the robustness of the process [6,7]. Meanwhile, the poorly designed production planning of steels and heating patterns for hot rolling can induce several problems during the process as follows:

- To achieve temperature uniformity in the discharged billet, the residence time of the billet tends to increase in the reheating furnace. In such cases, the reheating furnace can become a bottleneck in the rolling process, leading to a decrease in the productivity of the mill [1].
- The increased residence time of the billet increases the oxide scale formation on the billet surface, resulting in a high scale loss, and eventually leading to a decrease in the productivity of the mill [8].
- The setting of a high gas temperature in the reheating furnace to ensure the target temperature with acceptable uniformity in the discharged billet induces the distortion of the billet in the reheating furnace due to the temperature deviations of the billet within the region [9].
- The temperature deviations of the billet with region during the aforementioned heating process induce thermal cracks in the billet owing to thermal stress, potentially leading to the fracture of the billet in the reheating furnace [10].

**Figure 1:**
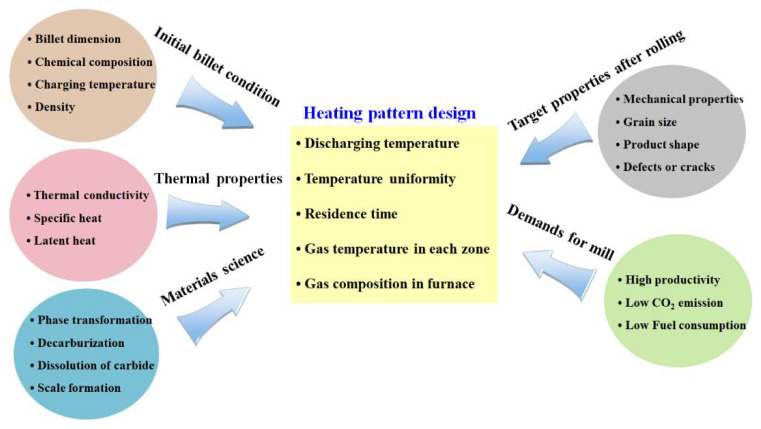
Design factors affecting the heating pattern of the billet/slab in an industrial hot rolling mill.

Based on the problems mentioned above and a literature review [2,11,12,13,14], the heating pattern of a billet needs to be designed from the perspectives of both thermal engineering and materials science. Based on the experiences of the author, the heating behaviors of carbon steels and austenitic stainless steels (STSs) were different despite their similar heating patterns. In a similar heating pattern, it was observed that the STSs were heated quickly as a whole heating stage and the temperature uniformity for each region of a billet was high when the billet was discharged. The author believes that these phenomena are closely related to the thermal properties of the steel because carbon steels and STSs have different thermal properties [15,16]. For example, Peet et al. [15] predicted and compared the thermal conductivity (*k*) of carbon steels and STSs based on the neural network technique. They showed that the *k* of carbon steels was much higher than that of STSs, indicating that the thermal behavior of two groups of steel was different during heating or cooling. These results suggest that the heating pattern should be different depending on the thermal properties of steels. Figure 1 summarizes the design factors affecting the heating pattern of an industrial rolling mill.

Among all of the design factors, the two steels have different *k* [17,18] and latent heat by phase transformation (*H*_latent_) [19,20]. That is, the *k* of carbon steels is higher than that of STSs, and carbon steels undergo a phase transformation during the heating process, whereas STSs have no phase transformation. The heating patterns of carbon steels and STSs might be different for obtaining the optimum thermal behavior in the reheating furnace. However, the influences of the thermal properties on the heating behaviors of the billet were hardly considered owing to the relatively low heating rate of the billet in the reheating furnace. In contrast, several studies were conducted regarding the influences of thermal properties of steels on the thermal behaviors during the cooling process because the cooling rate is relatively high, resulting in different cooling behaviors with respect to the thermal properties of metals [21,22]. Although the thermal properties of metals can slightly affect the average heating behaviors, they can affect the temperature deviation of the billet within the region owing to the mass effect of the material. In more detail, it takes time for the radiation energy delivered to the billet’s surface by the gas and wall in a reheating furnace to penetrate into the billet through conduction due to the large thickness of a billet. The time delay of heat transfer in a billet during heating owing to the mass effect of a billet can lead to a temperature difference in the billet. It is known that this time delay of the heat transfer in materials is strongly dependent on the thermal properties of the material.

The limited information available on the effects of *k* on the temperature distribution of the steel billet during the heating process drove the author to study this topic. Therefore, the author investigated the influences of *k* on the thermal behavior of a billet during the heating process to provide process engineers in a hot rolling mill with sufficient data to choose the optimal design conditions for a reheating furnace, which is an innovative feature of the present study. Two steels of medium-carbon steel and austenitic STS with completely different material properties such as *k* and *H*_latent_ were compared based on a numerical analysis.

This article analyzed the temperature profile of steels depending on the material properties using the temperature prediction model previously developed and verified by the author [23]. In particular, by comparing the carbon steel and STS, the different thermal behaviors of the two steels were evaluated. Finally, some suggestions for the gas temperature control for an industrial reheating furnace in a hot rolling mill are presented.

## 2. Numerical Model

### 2.1. Governing Equation

**Figure 2:**
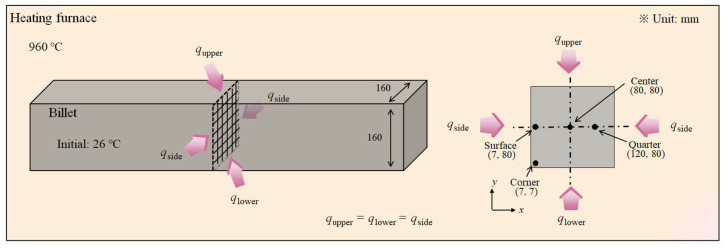
Schematic of the physical domain for a numerical simulation used in this study.

Generally, it is necessary to solve the full Navier–Stokes equation, energy conservation equation, and species equation governing the fluid flow and combustion in the furnace based on three-dimensional (3D) computational fluid dynamics (CFD) [1,24]. Although this 3D CFD analysis can offer thermal behaviors with a hot fluid flow and combustion within the entire furnace, several difficulties in treating the governing equations and complex furnace structures exist. For example, in order to simulate the entire structure of a heating furnace, a fairly large number of elements should be included in a physical domain. Accordingly, this approach has a long computational time and high costs, resulting in limits to its implementation in an industrial heating furnace. In addition, most industries are more interested in the temperature distribution of a billet rather than fluid flow within the heating furnace. Therefore, the thermal behavior of the 160 mm × 160 mm square billet was simulated using a two-dimensional (2D) finite volume method (FVM) as shown in Figure 2 in this study. Since the temperature deviation along the longitudinal direction of the billet was lower than that along the thickness direction, the heat transfer along the longitudinal direction of the billet can be ignored during heating. On the other hand, we should consider the heat transfer along the thickness direction of the billet due to the shape of square billet. Therefore, the 2D analysis is necessary to analyze the thermal behavior of the billet during the heating process. In this case, the transient Fourier heat conduction can solve the problem based on the conservation of energy in 2D rectangular coordinates as follows [24,25]:

(1) $$\rho c_{p}(T)\frac{\partial T}{\partial t}=\frac{\partial }{\partial x}\left(k(T)\frac{\partial T}{\partial x}\right)+\frac{\partial }{\partial y}\left(k(T)\frac{\partial T}{\partial y}\right)$$

where, *ρ*, *c*_p_, and *T* are the density, specific heat, and temperature of the billet, respectively. *t* is the time, and *x* and *y* are the coordinates of the 2D domain. *k* and *c*_p_ are variables depending on temperature.

### 2.2. Thermal Properties

**Figure 3:**
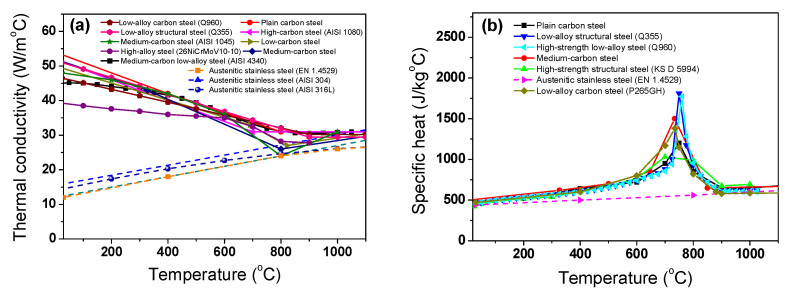

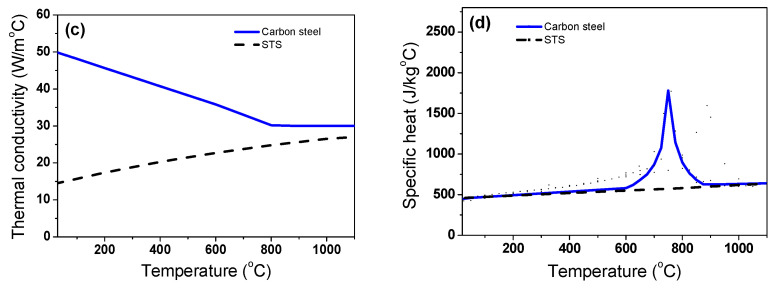
Comparison of the (**a**) thermal conductivity and (**b**) specific heat of the steels from Refs. [17,18,19,20,22,26,27,28,29,30,31,32,33]. The (**c**) thermal conductivity and (**d**) specific heat selected in this study.

The *k* and *c*_p_ of the billet are dependent on the temperature. In this study, the *k* and *c*_p_ of the billet were obtained based on the results of the literature review. Figure 3a,b show the *k* and *c*_p_ of the steels with temperature from several Refs. [17,18,19,20,22,26,27,28,29,30,31,32,33].

An abrupt change in the thermal properties appeared in the carbon steels due to the structure changes of the steels from the body centered cubic (BCC) to face the centered cubic (FCC) structures [29,34]. In carbon steels, *k* tends to decrease with an increasing temperature. In the case of the STS, *k* gradually increases with temperature. It is known that the thermal properties of metals vary with the composition, grain size, and cold deformation of the metal [35]. In this study, an alloyed medium-carbon steel (AISI 4137) and austenitic stainless steel (SUS304) were adopted for the test metals because it is widely used in wire, rod, and bar products. The average thermal properties of plain carbon steels and STSs were used as shown in Figure 3c,d, because this study mainly focuses on the effect of thermal properties on the temperature distribution of the billet during the heating process. The *k* and *c*_p_ were numerically implemented in Equation (1) as a function of temperature. Meanwhile, the density of the steel billets was assumed to be constant at 7850 kg/m^3^ [36,37].

### 2.3. Boundary and Initial Conditions

A boundary condition is the heat transfer way between the billet and heating furnace. The boundary conditions of the heating billet are the mixed conditions of convective (*q*_cv_) and radiative heat fluxes (*q*_r_) as follows [30]:

(2) $$-k\frac{\partial T}{\partial n}=q_{t}=q_{cv}+q_{r}$$

where *n* is the outer normal boundary surface and *q*_t_ is the total heat flux. *q*_cv_ and *q*_r_ are calculated as follows:

(3) $$q_{t}=h_{cv}\left(T_{g}-T_{s}\right)+\varepsilon \sigma \left(T_{g}^{4}-T_{s}^{4}\right)$$

where *h*_cv_ is the convection heat transfer coefficient, *ε* is the emissivity, and *σ* is the Stefan Boltzmann constant. *T*_g_ and *T*_s_ are the gas temperature in the heating furnace and surface temperature of the billet, respectively. Since approximately 90% of the heat flux to the billet depends on radiation in a furnace [30], the radiative heat transfer between the billet and environmental gas was considered, but the convection heat transfer was indirectly considered using an emission factor (*φ*) as follows:

(4) $$q_{t}=\varphi \sigma \left(T_{g}^{4}-T_{s}^{4}\right)$$

*T*_g_ is 960 °C throughout the study and varies depending on the gas temperature settings in Section 3.3. The small influence of convective and conduction heat transfers and several geometric factors for radiative heat transfer can be considered by using *φ*. In other words, the *φ* used in this study was a tuning parameter depending on several factors. We can predict the temperature distribution of the billet with the low computational cost and time when *φ* is accurately determined [3,4,5] compared with the 3D CFD analysis. In industrial fields, therefore, many hot rolling mills use these types of models to predict the temperature distribution of the billet in the reheating furnace. The disadvantage of this approach is that temperature measurement tests are strongly necessary to confirm and/or modify the *φ* values. The gas temperature around the billet in the furnace was assumed to be constant in this study. Therefore, *q*_t_ in Equation (4) was assumed to be the same on the four sides of the billet, as shown in Figure 1. That is, the upper, lower, right, and left sides of the billet had the same heat flux and *φ* value.

The temperature of the initial billet was assumed to be uniform at 26 °C prior to charging into the furnace, which is also a value measured via thermocouples. Therefore, the initial condition at *t* = 0 can be described as follows:

*T*(*x*, *y*, 0) = 26 °C (5)

### 2.4. Numerical Method and Determination of φ

The transient heat conduction equation in Equation (1) was discretized in *x*, *y*, and *t* using the 2D in-house FVM Fortran code by incorporating a central difference and implicit schemes. The discretized equations were iteratively solved using the tridiagonal matrix algorithm until the temperature contour within the billet satisfied a convergence criterion, which is well described by Patnakar [38]:

(6) $$max\left(\frac{\left|T_{i,j}-T_{i,j}^{old}\right|}{T_{i,j}}\right)\leq 10^{-6}$$

where $T_{i,j}$ and $T_{i,j}^{old}$ are the present and previous iteration values in the same time level, respectively. After a grid convergence test, the mesh system of 160 × 160 was adopted in this study.

The value of *φ* can be determined by comparing the calculated billet temperatures with the measured billet temperatures because *φ* is a tuning factor to improve the accuracy of the temperature prediction model in this study. In Ref. [23], the author conducted a temperature measurement test of the billet to determine *φ* during heating, and to evaluate the accuracy of the present temperature prediction model using a laboratory electric heating furnace. In other words, the temperature prediction model was validated using the heating test of a billet. The electric heating furnace was heated to 960 °C, then the AISI 4137 cold billet with a square cross-section of 160 mm × 160 mm and a length of 600 mm was charged into the furnace. The temperatures of four regions of the billet were measured using K-type thermocouples with 3.2 mm-diameter: the center, quarter, surface, and corner regions, as shown in Figure 2. The *φ* was determined by comparing the simulation temperatures with the measured billet temperatures at the corner region as a function of the residence time. The predicted temperatures of the four regions using *φ* exhibited reasonable agreement with the measured values. Although the derived *φ* values were varied in terms of residence time in the furnace, the average *φ* was approximately 0.5. A detailed description of the thermal model was well provided in Ref. [23]. The 2D temperature contours of the billet were obtained as a function of the residence time during heating with the values of *k*, *c*_p_, and *T*_g_ changing according to the analysis conditions.

## 3. Results and Discussion

### 3.1. Influence of Thermal Conductivity

**Figure 4:**
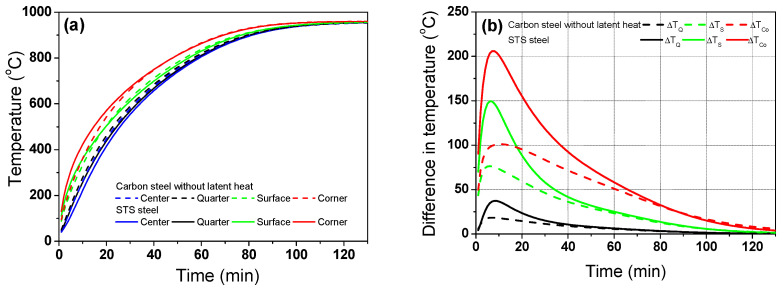
Comparison of the (**a**) temperature profiles and (**b**) temperature differences based on the center region between the carbon steel without latent heat and STS.

Figure 4a shows a comparison of the temperature profiles between the STS and carbon steel based on the different *k* in Figure 3c, aiming to evaluate the effect of *k* of the billet on the heat transfer mechanism within the billet. *T*_g_ is 960 °C during heating.

In carbon steel, no *H*_latent_ was assumed in this section to compare the single effect of *k* of the billet. In other words, it was assumed that the *c*_p_ in carbon steel is same as the *c*_p_ in STS. *H*_latent_ generated by the phase transformation during heating is an endothermic reaction in plain carbon steels. The peak in the *c*_p_ curve in Figure 3d is due to the enthalpy difference of BCC and FCC structures by phase transformation. And the different *c*_p_ values between plain carbon steel and STS (Figure 3d) affected the heating behaviors of the two billets based on Equation (1). *H*_latent_ was numerically calculated based on an inserted *c*_p_ value in Equation (1). For example, STS has no *H*_latent_ due to the flat profile of *c*_p_ with the temperature. Overall, the presence or absence of phase transformation during heating can affect the thermal behavior of billets. Without considering *H*_latent_, it is difficult to accurately predict the temperature profile of the steels during heating. In this section, no *H*_latent_ is assumed in the two steels in order to only evaluate the influence of *k* on the thermal behavior of steels during heating. The STS exhibited a higher temperature in the corner region and a lower temperature in the center region relative to the carbon steel, leading to a higher temperature deviation with the region compared with that of the carbon steel as shown in Figure 4b. The temperature deviation (Δ*T*) in each region was calculated by subtracting the center temperature (*T*_C_) from each point such as the quarter (*T*_Q_), surface (*T*_S_), and corner (*T*_Co_). For example, Δ*T* in the quarter region was calculated as follows:

Δ*T*_Q_ = *T*_Q_ − *T*_C_ (7)

The temperature deviation of the STS was quite high in the initial stage of heating owing to the high radiative heat flux in this stage stemming from the large temperature difference between the gas/wall temperatures in reheating the furnace and the surface temperature of the billet. The maximum temperature deviation of the STS was approximately 200 °C in this initial heating stage, while that of the carbon steel was approximately 100 °C. The low *k* of STS compared with that of the carbon steels (Figure 3c) resisted the heat conduction from the surface to the center of the billet, which can also be explained using the concept of a Biot number (*Bi*). That is, the STS had a higher *Bi* owing to the lower *k* compared with the carbon steel, leading to the higher temperature deviation in the billet with the region. Meanwhile, in the later stage of heating, it can be seen that the difference in the temperature deviation between the two steels was reduced because the heat flux by the external radiation was reduced in both steels in the later heating stage stemming from the small temperature difference between the furnace gas and billet surface in this heating stage, which can reduce the *Bi* of the both steels.

**Figure 5:**
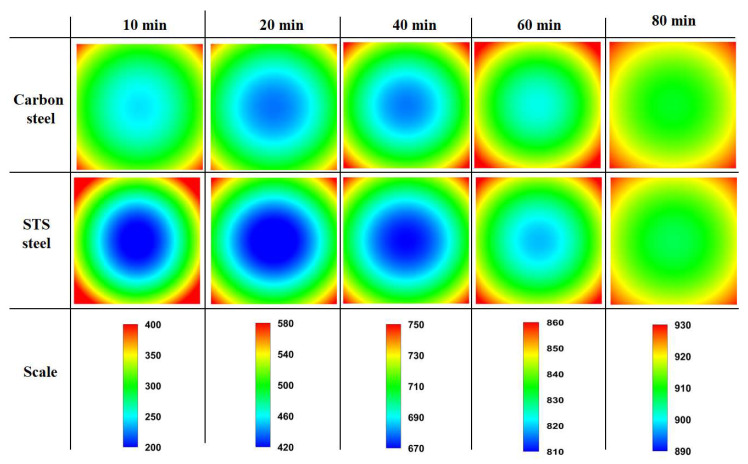
Comparison of the temperature contour (°C) of the billet between the carbon steel without latent heat and STS as a function of residence time.

Figure 5 compares the temperature contours of the billet between the carbon steel without *H*_latent_ and STS as a function of residence time. The post-processing for the temperature contour was performed with Tecplot (2014).

**Figure 6:**
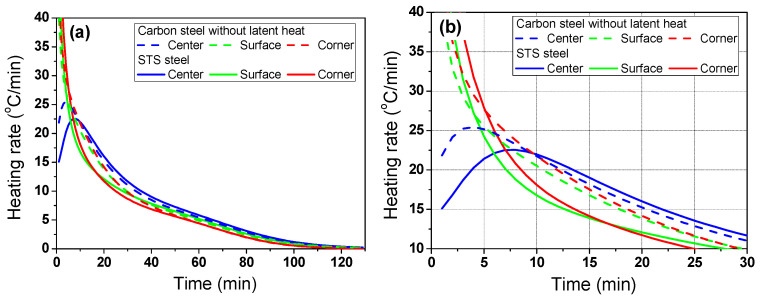
Comparison of the heating rate of the billet between the carbon steel without latent heat and STS with the region: (**a**) full and (**b**) initial ranges.

Clearly, the center temperature of STS was lower than that of carbon steel. In particular, the temperature deviation of the STS was higher at the initial stage of heating owing to the high heating rate (*R*_heat_) or *Bi* of the billet as shown in Figure 6. The *R*_heat_ was obtained using the temperature difference at a fixed element with a time step (Δ*t*) as follows:

(8) $$R_{heat}=\frac{T_{i,j}^{t+∆t}-T_{i,j}^{t}}{∆t}$$

In addition, the difference in *R*_heat_ in the STS was higher within the region compared to the carbon steel owing to the lower *k*. The temperature deviations of the STS and carbon steel were similar at the residence time of 80 min owing to the small *R*_heat_ of the two steels and the sufficient time for conductive heat transfer within the billet compared with the external incoming radiation.

### 3.2. Comparison of Thermal Behaviors of Carbon Steel and STS

**Figure 7:**
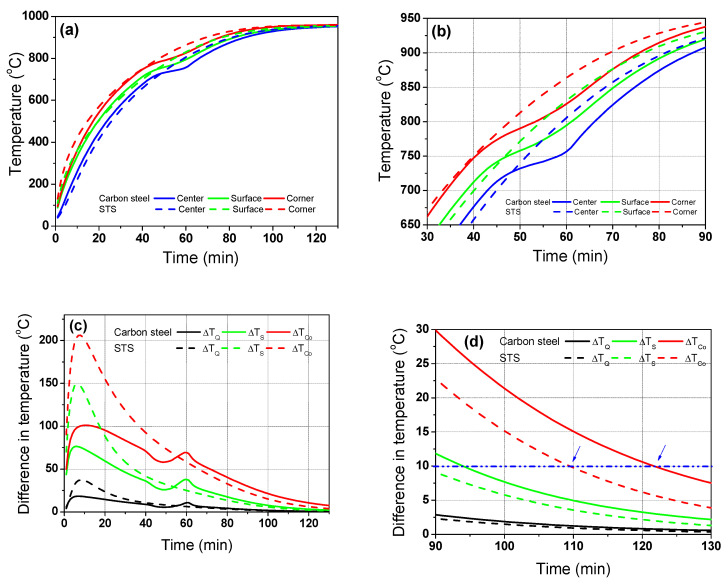
Comparison of the (**a**,**b**) temperature profiles and (**c**,**d**) temperature differences based on the center region between the carbon steel and STS. The (**b**,**d**) are the enlarged figures of (**a**,**c**), respectively.

According to the main results based on the numerical simulations, the temperature distribution of the billet during the heating process depended on the phase transformation and *k* of the steels, indicating that the carbon steel has a phase transformation with a relatively high *k* and STS has no phase transformation with a relatively low *k* can exhibit different heating behaviors during the heating process. Therefore, it is necessary to design the different heating patterns for the materials. Figure 7 compares the temperature profiles and corresponding temperature deviations between the carbon steel and STS at the furnace temperature of 960 °C, i.e., *T*_g_ is 960 °C.

At the initial stage of heating, the temperature deviation of the STS was higher than that of the carbon steel (Figure 7c) owing to its lower *k*. However, the temperature deviation of the carbon steel was higher than that of the STS when the phase transformation occurred in the carbon steel. Figure 7d compares the temperature deviations between the carbon steel and STS at the final stage of heating. If the acceptable temperature deviation along the thickness direction of the billet is 10 °C, we can reduce the residence time of the billet when using STSs. In other words, the residence time of the STS was approximately 110 min and that of the carbon steel was approximately 122 min, which means that the phase transformation increased the temperature deviation of the billet in the later stage of heating, leading to an increase in the residence time.

**Figure 8:**
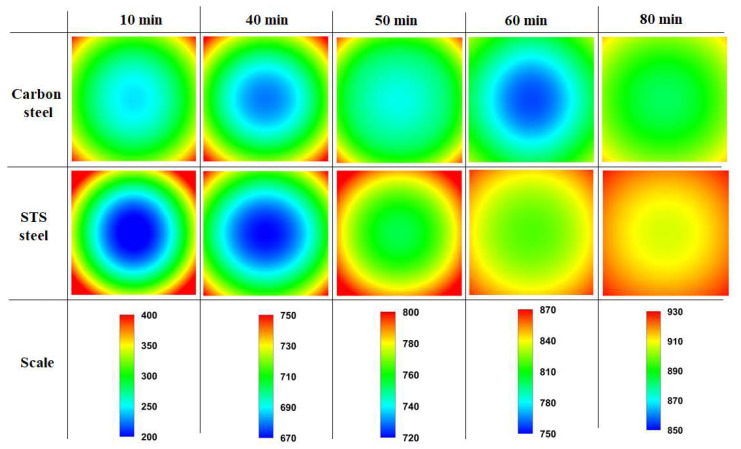
Comparison of the temperature contour (°C) of the billet between the carbon steel and STS as a function of residence time.

Figure 8 shows a comparison of the temperature contours of the billet between the carbon steel and STS as a function of residence time. As mentioned above, the temperature deviation of the STS billet was higher in the initial heating stage, and that of the carbon steel billet was higher after the phase transformation.

**Figure 9:**
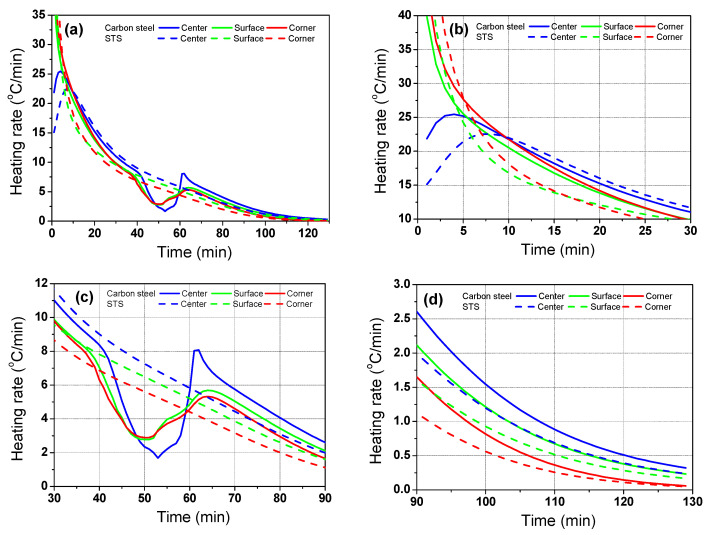
Comparison of the heating rate of the billet between the carbon steel and STS with the region: (**a**) full; (**b**) initial; (**c**) phase transformation; and (**d**) final ranges.

Figure 9 shows a comparison of *R*_heat_ of the billet between the carbon steel and STS with the region. The *R*_heat_ of the billet with region depended on the *k* of the billet in the initial heating stage, whereas it depended on *H*_latent_ after the phase transformation of the billet.

**Figure 10:**
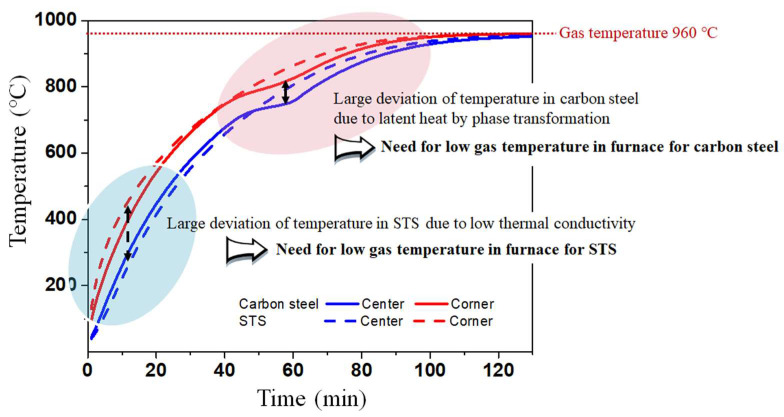
Schematic diagram showing gas temperature control considering thermal conductivity and latent heat by phase transformation in steels.

Overall, as summarized in Figure 10, the temperature deviation of the billet increased with decreasing *k* owing to the low conductive heat transfer within the billet, especially in the initial stage of heating owing to the higher radiative heat transfer from the gas/wall in the furnace to the surface of the billet. It means that we should decrease the gas temperature in the initial heating stage or preheating zone in a reheating furnace when heating the billet with low *k* such as STS and high-alloyed steels. Meanwhile, the temperature deviation of the carbon steel increased after the phase transformation. The phase transformation of steels generally occurred in the billet temperature range of 700 °C–800 °C, indicating that we should decrease gas temperatures in a reheating furnace in this temperature range of the billet. In summary, the different heating patterns are strongly necessary for carbon steel and STS billets.

### 3.3. Suggestion for Gas Temperature Control in Industrial Reheating Furnace

**Figure 11:**
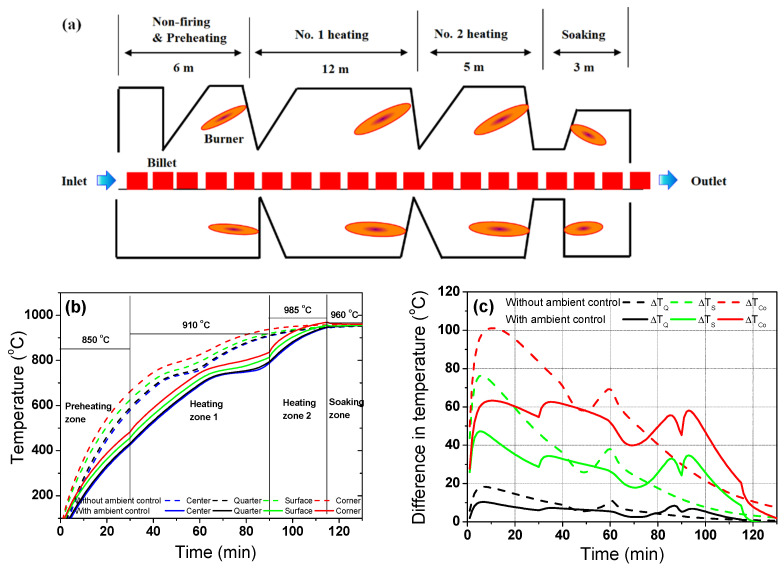
(**a**) Schematic description of arbitrarily designed walking-beam type reheating furnace for carbon steel. Comparison of the (**b**) temperature profiles and (**c**) temperature differences of the carbon steel with and without gas temperature control in the furnace. The temperatures presented in (**b**) indicate the gas temperatures of each zone in the reheating furnace.

It is known that the high temperature deviation of the billet/slab induces thermal stress during the heating process, leading to distortions and/or thermal cracks in the billet in the reheating furnace [9,10]. Sometimes, a billet was fractured from thermal cracks in the reheating furnace. Huang et al. [10] reported the fracture mechanism of the silicon steel slab in a reheating furnace and showed a strong influence of the heating rate on the fracture of the slab during heating. Accordingly, the industrial reheating furnaces for the hot rolling generally consist of four heating zones for controlling the gas temperature in the furnace: preheating, primary heating or no. 1 heating, secondary heating or no. 2 heating, and soaking zones [3,11,36]. The cold billet is transported step-by-step from the preheating zone to the soaking zone by a walking-beam or pusher [13]. In this section, we applied the conceptual idea for determining the heating pattern considering *k* and *H*_latent_ for carbon steel and STS to an industrial walking-beam type reheating furnace. In this study, other furnace operating conditions, such as the pitch time of the walking-beam, heat loss due to the skid button, billet spacing, burner position, and so on, were not considered except for the gas temperature in a furnace. In addition, the furnace dynamic behaviors due to combustion, fluid flow, and walking-beam were not considered in the study. Under these assumptions, the results obtained from the electric heating furnace were applied to understand the thermal behavior of a billet in an industrial walking-beam-type reheating furnace. Figure 11 shows an example of the temperature profiles and differences in the carbon steel as a function of the temperature control of each zone.

A reheating billet furnace of 26 m was arbitrarily defined, as shown in Figure 11a, based on the No. 8 wire rod mill at Kakogawa works in Japan [39]. The length of each zone of the reheating furnace was arbitrarily selected to apply the results of this study to industrial heating furnaces. It should be noted that the dimension of the reheating furnace for billets was generally smaller than that for slabs [24] and blooms [40] due to the small dimension of the cross-section of a billet.

Most of the radiation heat flux into the billet occurred at the initial stage of heating, as shown in Figure 9, while the billet received a little radiative heat flux from the gas in the later stage of heating. Therefore, it is important to distribute the heat flux homogeneously throughout the reheating process, especially during the phase transformation of a billet. The temperature deviation of the billet in the initial heating stage can be easily reduced by controlling the temperature of the preheating zone. That is, *Bi* is reduced by controlling the external radiative heat transfer coefficient because it is impossible to control the *k* of the billet. In addition, the temperature deviation of the billet in the final stage of heating can be controlled based on the gas temperatures in the secondary heating and soaking zones. That is, the temperature deviation of the discharging billet can be reduced by increasing the temperature in the secondary heating zone relative to the soaking zone. More importantly, a high heat flux should not be applied in the heating stage of the phase transformation in a billet because a phase transformation promoted the temperature deviation with a region of the billet [23]. For example, a relatively low gas temperature should be maintained in the primary heating zone. Based on these results, an arbitrarily new heating pattern was designed as listed in Table 1. And, the temperature in the billet was presented in Figure 11b using the new designed heating pattern, which is marked with ambient control on Figure 11b. In addition, temperature deviation within the region was compared in Figure 11c. Temperature deviation with the region was calculated using Figure 11b based on Equation (7). It can be seen that the temperature deviations at the initial heating stage and during the phase transformation are smaller in the case with ambient control compared to the case without ambient control.

**Figure 12:**
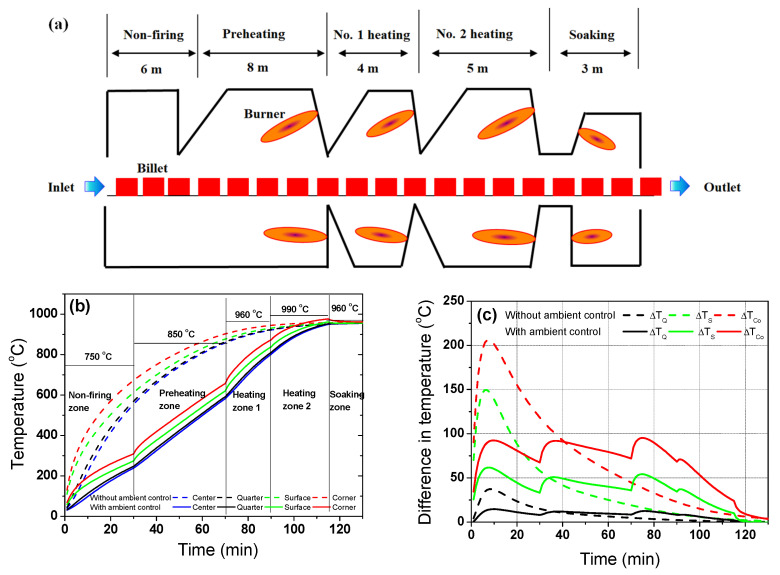
(**a**) Schematic description of arbitrarily designed walking-beam type reheating furnace for STS. Comparison of the (**b**) temperature profiles and (**c**) temperature differences of the STS with and without gas temperature control in the furnace. The temperatures presented in (**b**) indicate the gas temperatures of each zone in the reheating furnace.

Figure 12 also shows an example of the temperature profiles and differences for the STS in an arbitrarily designed reheating furnace (Figure 12a) as a function of the gas temperature control in the reheating furnace.

Since *k* of the STS was relatively low, the preheating zone or non-firing zone needed to decrease the temperature deviation of the STS billet in the initial stage of heating. For example, a low gas temperature was applied at the non-firing zone and preheating zone, as listed in Table 1. The temperature deviation within the region in the billet was dramatically reduced by applying a low gas temperature in the initial stage of heating the STS, as shown in Figure 12b,c.

Overall, the gas temperature in reheating furnace needs to be reduced in the initial stage of heating and during the period of the phase transformation to improve the uniformity of the billet temperature or to reduce the thermal stress in the billet. Therefore, a non-firing zone is strongly recommended for STSs or high-alloyed steels, as listed in Table 1 because it is known that the *k* of metals decreases with an increasing chemical composition [10,19]. In addition, the hot charging of a billet can reduce the temperature deviation or thermal stress in the billet [41] because it effectively decreases the *Bi* of the billet. Meanwhile, it is recommended that the phase transformations of carbon steels occur in the primary heating zone at a low temperature. However, the low temperature in the primary heating zone can increase the total residence time of the billet owing to the desired uniformity of the discharging billet. Therefore, the secondary heating zone needs to have a higher temperature than the soaking zone to reduce the residence time of the billet. In other words, each zone in the reheating furnace plays a specific role for the quality control of a billet, as summarized in Table 2.

The heating pattern of the non-firing and/or preheating zones should be designed in consideration of *k* of the billet, the size of billet, and the thermal stress. A billet charging temperature is also an important design factor because the temperature difference between the gas/wall in a furnace and the surface of a billet decreased with the increasing charging temperature of a billet. The heating condition in the primary heating zone should consider the *H*_latent_ of the billet. If possible, the heat flux should be set to small during the phase transformation of a billet to reduce the temperature deviation with the region of a billet during phase transformation. The secondary heating zone must consider the uniformity of the discharging billet and the total residence time during reheating. The gas temperature in this zone needs to be set to 10–60 °C higher than the target discharging temperature of the billet to ensure the temperature uniformity of the billet and to reduce the residence time. In addition, the formation of the oxide scale and decarburization in the surface of a billet and the austenite grain coarsening should be considered to design the heating pattern due to the high temperature in the secondary heating zone. Finally, the soaking zone considered the desired target temperature [42] and the dissolution of carbides or nitrides in the billet [43]. Additionally, to ensure the temperature homogeneity of a billet for subsequent hot rolling, the thermal loss of the billet by the skid system in the reheating furnace should be considered in this soaking zone [44]. Although similar heating patterns proposed in this study have been widely used in the industrial field [13,42], it is still being utilized with a lack of theoretical background. Therefore, when a problem occurs in the billet during the heating process in an industrial hot rolling mill, the above suggestions tend to be ignored, and a new heating pattern is designed without considering the phase transformation, *k*, and the temperature uniformity of the billet, leading to other issues regarding the billet quality.

Meanwhile, it is worth noting that the oxide scale formation on the billet surface affects the thermal behavior of the billet [25,45]. For example, the characteristics of the scale formation behaviors between the carbon steel and STS were significantly different, resulting in different thermal behaviors with the steels in the reheating furnace, leading to the necessity for the different heating patterns of the two steels. This study did not consider the influence of the oxide scale formation on the billet surface on thermal behaviors during heating, which is one of the ongoing research topics for the author including the reconfirmation of the present numerical study.

## 4. Conclusions

Based on a comparative study on the thermal behaviors of carbon steel and STS billets during heating, the following conclusions are induced.

1. The thermal conductivity affected the thermal behavior of the billet in the initial stage of heating due to the high temperature difference between the surface of the billet and the gas in the reheating furnace. In this case, the heat flux from the gas to the billet was high, originating from the radiative heat transfer mechanism and resulting in a high *Bi* in this heating stage.
2. A non-firing zone and/or a preheating zone with a low gas temperature are necessary to reduce the *Bi* of the billet, especially for the high-alloyed steels, including STSs, because the thermal conductivity of these steels was relatively low.
3. The phase transformation of the carbon steels needs to occur in the primary heating zone, and this zone needs to have a relatively low temperature to reduce the temperature deviation or thermal stress in the billet.
4. The heating pattern of the carbon steels and STSs in the reheating furnace should be designed differently considering the thermal conductivity and latent heat by phase transformation of the steels to obtain a high heating quality for the billet.

## Figures and Tables

**Table 1:** Comparison of the gas setting temperature for each zone in the heating furnace between carbon steel and STS. The target discharging temperature is 960 °C.

Steels	Zone in Reheating Furnace (Tg, °C)				
	Non-Firing	Preheating	No. 1 Heating	No. 2 Heating	Soaking
Carbon steels	Not necessary	850	910	985	960
Stainless steels	750	850	960	990	960

**Table 2:** Design factors and strategies in each zone of walking-beam type reheating furnace from a temperature control perspective.

Zone in Furnace	Design Factor	Strategy for Gas Temperature Control
Non-firing	• Thermal conductivity • Billet cross-section size (mass effect) • Initial billet temperature (cold or hot charge) • Thermal stress including high-temperature toughness of billet	• Necessary for the billet with large size and/or high alloyed steels including STS
Preheating		• Low temperature for high-alloyed steels including STS
Primary heating (No. 1 heating)	• Latent heat by phase transformation • Volume expansion by structural change • Thermal stress including the high-temperature toughness of the billet	• Low temperature for carbon steels with phase transformation
Secondary heating (No. 2 heating)	• Temperature uniformity of the discharged billet and residence time • Dissolution of carbides or nitrides • Formation of oxidation scale and decarburization	• Billet target temperature + (10~60 °C)
Soaking	• Target temperature of billet • Dissolution of carbides or nitrides • Grain size • Skid button effect • Formation of oxidation scale and decarburization	• Billet target temperature

## Data Availability

Data are contained within the article.
